# SARS-CoV-2 Variants of Concern and Variants of Interest Receptor Binding Domain Mutations and Virus Infectivity

**DOI:** 10.3389/fimmu.2022.825256

**Published:** 2022-01-27

**Authors:** Haolin Liu, Pengcheng Wei, John W. Kappler, Philippa Marrack, Gongyi Zhang

**Affiliations:** ^1^Department of Immunology and Genomic Medicine, National Jewish Health, Denver, CO, United States; ^2^Department of Immunology and Microbiology, School of Medicine, Anschutz Medical Center, University of Colorado, Aurora, CO, United States

**Keywords:** SARS-CoV-2, variants, infectivity, receptor binding domain, ACE2, bamlanivimab, vaccine

## Abstract

The severe acute respiratory syndrome coronavirus 2 (SARS-CoV-2) pandemic has lasted more than 2 years with over 260 million infections and 5 million deaths worldwide as of November 2021. To combat the virus, monoclonal antibodies blocking the virus binding to human receptor, the angiotensin converting enzyme 2 (ACE2), have been approved to treat the infected patients. Inactivated whole virus or the full-length virus spike encoding adenovirus or mRNA vaccines are being used to immunize the public. However, SARS-CoV-2 variants are emerging. These, to some extent, escape neutralization by the therapeutic antibodies and vaccine-induced immunity. Thus, breakthrough infections by SARS-CoV-2 variants have been reported in previously virus-infected or fully vaccinated individuals. The receptor binding domain (RBD) of the virus spike protein reacts with host ACE2, leading to the entry of the virus into the cell. It is also the major antigenic site of the virus, with more than 90% of broadly neutralizing antibodies from either infected patients or vaccinated individuals targeting the spike RBD. Therefore, mutations in the RBD region are effective ways for SARS-CoV-2 variants to gain infectivity and escape the immunity built up by the original vaccines or infections. In this review, we focus on the impact of RBD mutations in SARS-CoV-2 variants of concern (VOC) and variants of interest (VOI) on ACE2 binding affinity and escape of serum antibody neutralization. We also provide protein structure models to show how the VOC and VOI RBD mutations affect ACE2 binding and allow escape of the virus from the therapeutic antibody, bamlanivimab.

## Introduction

Since the first human positive case was reported at the end of 2019 (1), severe acute respiratory syndrome coronavirus 2 (SARS-CoV-2) has spread all over the world and become a global pandemic with more than 260 million infections and 5 million deaths as of the end of 2021 (WHO SARS-CoV-2 cases). To treat the newly infected persons, monoclonal antibodies (mAb) have been isolated from the B cells of SARS-CoV-2-infected patients and developed as drug candidates (2, 3). These mAb drugs, including bamlanivimab (4) and antibody cocktails such as casirivimab and imdevimab (5) showed promise in clinical trials in reducing viral loads during the early stage of infection. They have been granted emergency use authorization (EUA) by the US Food and Drug Administration (FDA) (6). All these EUA mAbs target the receptor binding domain (RBD) of the surface spike protein of SARS-CoV-2 and inhibit the ability of the virus to attach to host cellular receptor, angiotensin converting enzyme 2 (ACE2). Administration of the mAbs to the patients provides fast but temporary passive humoral immunity against the virus. Meanwhile, several types of SARS-CoV-2 vaccines, such as the traditional inactivated whole virus vaccines (7) and adenoviruses (8, 9) or mRNA vaccine encoding the viral spike (10, 11), have been developed and approved for use to immunize the uninfected people to help them build up long-term immunity against the virus. Although SARS-CoV-2 does not mutate as rapidly as other RNA viruses, its RNA-dependent RNA polymerase (RdRp) appears to be somewhat more error prone than the DNA polymerases in DNA viruses (12). Some of the mutations benefit the virus by increasing its infectivity. As a result, SARS-CoV-2 variants with such mutations replace the original virus and become the dominant strains. The spike protein D614G mutation is one such, that allowed this variant to replace the ancestral virus globally within 4 months from first appearance in February 2020 to dominance in the following June (13). Analysis showed that D614G mutation converted more spike RBD to a fusion-competent state, the so-called upper conformation (13).

Apart from the consequences of increased infectivity by variants created by the error rate of the RdRp, viral mutants may be selected by the preexisting immunity to the ancestral virus in previously infected individuals, or vaccinated individuals, or patients given protective mAbs (14, 15). This is unexpected in previously infected individuals since immunity against components of the virus other than its spike should be able to prevent variant escape from the preexisting immunity. However, more than 90% of the neutralizing antibodies in SARS-CoV-2 virus infected, convalescent patients target the spike RBD (16), and therefore spike variants may escape preexisting immunity in these individuals. The SARS-CoV-2 RBD is located between the N-terminal domain (NTD) of the S1 and S2 subunits of the spike protein and is composed of approximately 200 amino acids. It interacts with ACE2 by the receptor binding motif (RBM) which is formed by about 70 amino acids with 17 amino acids in direct contact with ACE2 (17).

**Figure 1:**
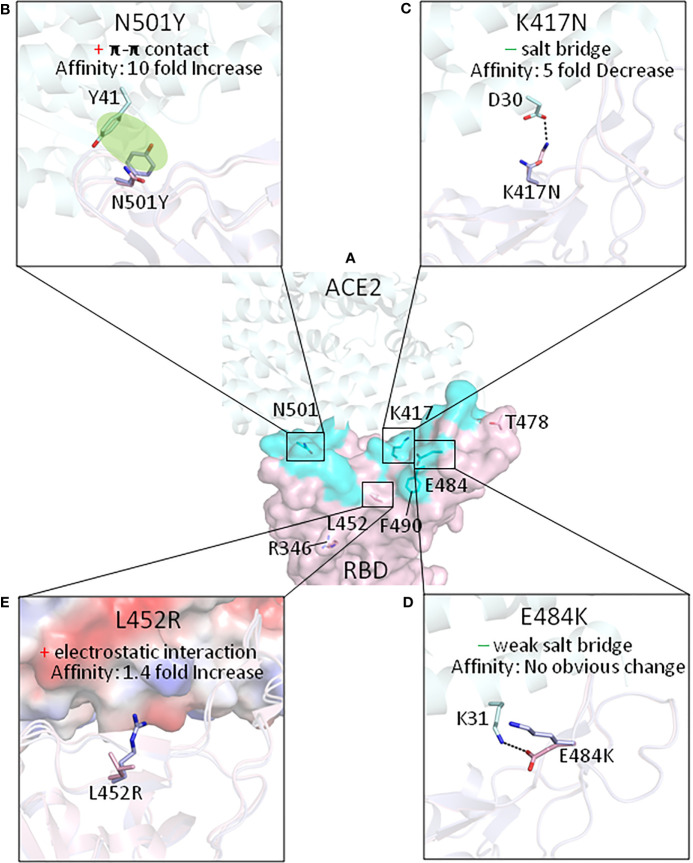
The effect of the mutations of RBDs on interactions with ACE2, for currently identified SARS-CoV-2 VOC and VOI (with the exception of the Omicron variant). **(A)** The amino acids which have been mutated in the RBD variants are shown on the ancestral RBD with ACE2 in light cyan. Key mutations that affect the affinity of the RBD for ACE2 affinity are shown in **(B)** N501Y mutation; **(C)** K417N mutation; **(D)** E484K mutation; and **(E)** L452R mutation. “+” means gain of interaction while “−” indicates loss of interaction. The protein structures we used for modeling are from PDB with the following ID. Ancestral RBD: 6M0J, Alpha variant RBD: 7EKG, Beta variant RBD: 7EKG, Delta Variant RBD: 7V8B.

**Figure 2:**
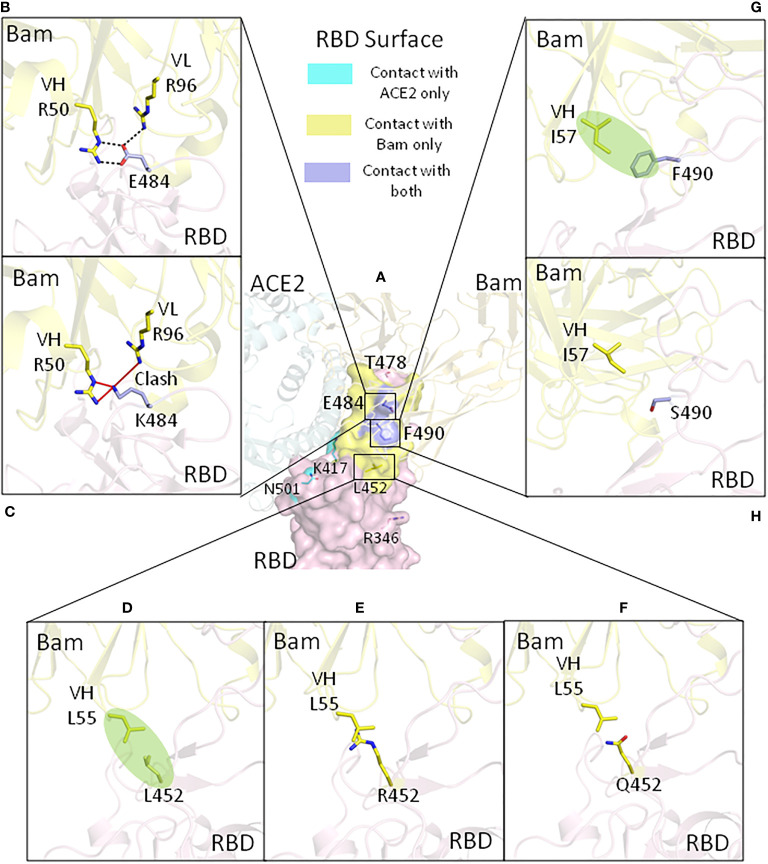
The effect of the mutations of RBDs on interactions with bamlanivimab for currently identified SARS-CoV-2 VOC and VOI (with the exception of the Omicron variant). **(A)** The amino acids which have been mutated in the variants are shown on the ancestral RBD overlaying with ACE2 and bamlanivimab. Key ancestral and variant mutations that affect bamlanivimab affinity are shown in the following: **(B)** for ancestral glutamic acid 484, **(C)** for E484K mutation, **(D)** for ancestral leucine 452, **(E)** for L452R, **(F)** for L452Q mutation, **(G)** for ancestral phenylalanine 490, and **(H)** for F490S mutation. Bam, bamlanivimab. We used 7KMG (PDB ID) for bamlanivimab and RBD modeling.

Currently, WHO has five SARS-CoV-2 variants of concern (VOC), Alpha, Beta, Gamma, Delta, and Omicron, and two SARS-CoV-2 variants of interest (VOI), Lambda and Mu (WHO SARS-CoV-2 Variants). These variants affect the binding of the viral RBD to ACE2, and/or the infectivity of the virus, and/or neutralization of the virus by mAbs to different degrees while maintaining the overall structure of the spike (**Table 1**) (18–22). The three RBD mutations, for example, in the Beta, Gamma, or Mu variants do not change the overall structure of the RBD from the ancestral version (**Figure 1A**) (22) and a low root-mean-square deviation (RMSD) between the Beta variant RBD and the ancestral RBD of 0.62 indicates the three mutations have little effect on the overall backbone of RBD. However, they do affect the binding of at least one of the mAbs since its target on the RBD overlaps with a mutated amino acid (**Figure 2A**). Currently, the broadly neutralizing antibodies targeting the RBD region have been divided into four classes depending on where the antibodies bind (23). In general, because the existing mAb drugs and the vaccines were developed against the ancestral spike, they may be less effective against the current and future VOC and VOI, especially with the recent surge of the Delta and predicted surge of the newly discovered Omicron variant thus allowing breakthrough infections in previously infected and fully vaccinated persons (24).

**Table 1:** SARS-CoV-2 VOC and VOI RBD mutations on ACE2 or bamlanivimab affinity.

Variant category	Variants (nomenclature)	Mutations in RBD region	ACE2 affinity change versus ancestral RBD	Bamlanivimab affinity change versus ancestral RBD
Variant of concern (VOC)	Alpha	N501Y	10-fold increase	No change
	Beta	K417N E484K N501Y	2-fold increase	E484K abolishes mAb binding
	Gamma	K417T E484K N501Y	5-fold increase	E484K abolishes mAb binding
	Delta	L452R T478K	2-fold increase	L452R decreases mAb by 20-fold
	Omicron	G339D S371L S373P S375F K417N N440K G446S S477N T478K E484A Q493K G496S Q498R N501Y Y505H	Potential increase due to N501Y mutation and/or other ACE2 contact residue mutations	E484A abolishes mAb binding
Variant of interest (VOI)	Lambda	L452Q F490S	No change	L452Q decreases mAb by 8-fold F490S decreases mAb by 20-fold
	Mu	R346K E484K N501Y	9-fold increase	E484K abolishes mAb binding

We have been closely monitoring the effects of mutations in the RBD region of VOC or VOI on ACE2 binding and antibody escape. We measured the affinity of human ACE2 to the ancestral and VOC or VOI RBDs using surface plasmon resonance (SPR) with biotinylated birA-tagged human ectodomain ACE2 immobilized on the Biacore streptavidin chip as ligand and RBD as analyte (18–20). Since ACE2 binds RBD with its amino terminal domain (17), the carboxyl terminal biotinylation of ACE2 does not interfere with its interaction with RBD. Biotin immobilization of ACE2 on the streptavidin chip maintains the coated ACE2 in the native form, and the analyte RBD in the solution is likewise fully native. With the same ACE2 chip, our method would accurately measure and compare the affinity between ACE2 and RBD of VOC and VOI. It is noteworthy that different binding affinity values of SARS-CoV-2 RBD to ACE2 have been reported by different groups in the world. Several factors may lead to this discrepancy. First of all, different groups may use different platforms to measure the affinity. Among different technologies, SPR-based affinity measurement remains the gold standard for the affinity measurement with real-time on and off rates. Secondly, ensuring the native form of ACE2 and RBD and keeping the ligand constant are the keys to determine the accurate affinity value and compare the effect of RBD mutations on ACE2 binding. Last, the affinity value also depends on what kinds of methods researchers use to determine the analyte concentration. It is important to use the same method for both ancestral and mutant RBD to draw the right conclusion. Meanwhile, since the RBD contains multiple B-cell epitopes, we used the well-characterized therapeutic mAb, bamlanivimab, as a model to elucidate how the RBD of VOC or VOI affect the mAb binding. Due to the relative high stability of immunoglobulin IgG protein, we coated the mAb directly on the CM5 chip with the amine coupling method as ligand and RBD as analyte (18). Again, the same bamlanivimab-coated chip provided accurate affinity measurement and comparison of antibody affinity to different RBD. In this review, we will dissect how the mutations in the RBD region of VOC or VOI affect ACE2 binding and escape the bamlanivimab response by solid experimentation measuring the binding affinity and also providing structure modeling. Our findings and that of others are described below.

### Variants of Concern

The SARS-CoV-2 VOC is a variant with genetic changes that would increase its transmission or immune escape and threat the global health (WHO SARS-CoV-2 Variants). As of November 2021, there are five variants of concern of SARS-CoV-2. They are named Alpha, Beta, Gamma, Delta, and Omicron, each with different mutations within the RBD region. More knowledge is accumulating about the properties of the newly discovered Omicron variant. Knowledge about each variant is listed below.

#### Alpha Variant

First identified in the UK in September 2020, the Alpha variant became the dominant strain in the UK at the end of January 2021 (Wellcome Sanger Institute). As of December 10, 2021, the total reported number of infections by Alpha variant in the UK is 277,468 (U.K. Health Security Agency). Of interest for this review, the Alpha variant has an asparagine to tyrosine mutation at the 501 amino acid site in RBD region (N501Y). The original asparagine is a contact residue with ACE2, forming a hydrogen bond with tyrosine 41 (Y41) of ACE2. The N501Y mutation lost this hydrogen bond; however, it created a much stronger interaction *via* π-π stacking, formed between 501Y of spike and 41Y of ACE2, and also cation-π interaction between 501Y and lysine 353 of ACE2 (**Figure 1B**) (22). These newly formed interactions increased ACE2 binding affinity by 10-fold compared with the ancestral RBD, contributed by a 1.3-time faster association rate and a 7.7-time slower dissociation rate (18). This 10-fold increase of RBD/ACE2 binding affinity is the major factor making the Alpha variant more contagious than the ancestral strain. Also, the N501Y-mutated SARS-CoV-2 spike functioned in mice, largely due to the increase of binding affinity to mouse ACE2 (25).

Fortunately, neither asparagine nor tyrosine at amino acid 501 of the spike comprises part of the epitope of bamlanivimab, and therefore, the N501Y mutation does not affect engagement of this mAb (**Table 1**). The affinity of bamlanivimab for the ancestral or Y501-RBD is around 0.8 nM. The affinity of ACE2 for ancestral or Y501-RBD is at 5.8 and 0.57 nM, respectively (25). Thus, affinity of the Y501-RBD for ACE2 is higher than that of bamlanivimab and RBD, indicating that more mAb might be needed to treat Alpha variant versus ancestral virus-infected patients. (Note that these numbers do not take into account the avidity of the polyvalent virus versus the divalent antibody for their RBD targets). This is also true for convalescent or vaccine-induced serum antibody. Such antibody neutralized a pseudovirus expressing the Alpha variant spike 2.3 times less well than a similar virus expressing the ancestral spike (26). Ninety-five percent of the memory B cells induced by the mRNA vaccine against the ancestral RBD recognize Y501-RBD (27). Therefore, the decrease in antibody effectiveness against the Alpha variant is mainly due to the increase in the affinity of the Y501 spike for ACE2.

#### Beta Variant

The Beta variant was first identified in South Africa in May 2020 and became the dominant strain there (WHO SARS-CoV-2 Variants). There are three mutations in the RBD region, K417N, E484K, and N501Y. The effects of the N501Y mutation are discussed in the section on the Alpha variant above.

The SARS-CoV-2 spike 417 lysine is a contact residue with ACE2 (17). Its positively charge side chain forms a tight salt bridge (at 2.9 Å in distance) with the negatively charged side chain of aspartic acid 30 of ACE2 (19). The lysine mutation to asparagine, causes the loss of the original salt bridge interaction (**Figure 1C**), and therefore, the K417N mutation, if alone, decreases the ACE2 affinity by fivefold (19, 21).

Since neither lysine nor asparagine at spike 417 is part of the bamlanivimab epitope, the K417N mutation did not affect bamlanivimab binding. However, this mutation has been shown to affect the engagement of other antibodies, including etesevimab (28), an antibody that FDA approved for use in combination with bamlanivimab to treat COVID-19 patients.

The 484 glutamic acid in SARS-CoV-2 spike protein is not a contact residue with ACE2 (17). The ancestral negatively charged glutamic acid at this position forms a weak salt bridge (at 4.4 Å in distance) with positively charged lysine 31 of ACE2. The spike E484K mutation causes a loss of this weak salt bridge (**Figure 1D**). However, since this interaction was relatively weak due to the long distance, losing this interaction would probably have no obvious effect on the affinity of spike for ACE2 (19). The major effect of the E484K mutation is that it promotes virus escape from broadly neutralizing antibodies. E484, for example, forms three strong salt bridges with arginines on bamlanivimab (**Figure 2B**). When mutated to K484, the positively charged lysine would repel the positively charged arginines and thus fully abolish the binding of bamlanivimab (**Figure 2C**; **Table 1**) (19). Therefore, bamlanivimab fails to protect human patients or human ACE2 transgenic mice against infection by SARS-CoV-2 variants harboring the E484K spike mutation (14, 29). It was also found that the E484K mutation decreases binding by convalescent plasma polyclonal antibodies by more than 10-fold in certain human subjects (30).

When these three mutations exist together as in the Beta variant RBD, the combination of the effects of N501Y and K417N increases the affinity of the RBD for ACE2 by 2-fold compared with that of the ancestral RBD (**Table 1**). This is the partial reason why the Beta variant was the dominant strain in South Africa. Other reasons may stem from immune escape caused by the E484K and K417N mutations. Pseudovirus with the Beta variant spike decreased mRNA-vaccinated serum antibody neutralization by 7.6-fold (26). More than 20% of memory B cells against the ancestral RBD in the mRNA-immunized human subjects have decreased binding to the Beta variant RBD (27).

#### Gamma Variant

The Gamma variant was identified in Brazil in November 2020. The RBD of the Gamma variant is similar to that of the Beta variant, with the exception that K417 has been mutated to threonine instead of asparagine (WHO SARS-CoV-2 Variants). The K417T mutation alone decreased ACE2 affinity by 3-fold; however, this loss was accommodated by the accompanying E484K and N501Y mutations resulting in improved affinity for ACE2 of the Gamma variant RBD by 5-fold (**Table 1**) (21). Like the K417N mutation, K417T did not affect bamlanivimab binding. As expected, the positively charged side chain of lysine to polar threonine side chain mutation would also change the binding of antibodies targeting around this area. Pseudovirus with the Gamma variant spike decreased mRNA-vaccinated serum antibody neutralization by 2.9-fold (26).

#### Delta Variant

The Delta variant was first identified in India in October 2020 and then became the dominant strain all over the world (WHO SARS-CoV-2 Variants). In the UK, only 3 months, from April to June 2021, were needed for the Delta variant to replace the previously dominant Alpha variant (Wellcome Sanger Institute). In contrast to the Alpha variant, the total reported number of infections by Delta variant in the UK is 1,548,561 as of December 10, 2021 (UK Health Security Agency). The Delta strain is well known for its higher virus load and shorter incubation period in infected human subjects. It has been reported that the virus load of the Delta variant is 1,000 times higher than the ancestral strain (31). Among the mutations on the spike, the P681R mutation at the furin cleavage site, accounts for higher cleavage rate between S1 and S2 domain of spike, leading to easier entry of the virus into cells (32).

The Delta variant RBD contains L452R and T478K mutations and increased ACE2 affinity by 2-fold (**Table 1**) (20), which also plays a role in higher infectivity of the Delta variant. Reduced serum antibody neutralization of the Delta variants has also been reported (33), and pseudovirus with the Delta variant spike decreased mRNA-vaccinated serum neutralization by 2.9-fold (26).

Leucine 452 of the ancestral strain is not the contact residue with ACE2 (17). When mutated to arginine, the RBD affinity for ACE2 is increased 1.4-fold compared with the ancestral RBD (20). This may be because R452 can form a tighter electrostatic interaction with the negatively charged patch involving the glutamic acid and aspartic acid on the ACE2 surface (**Figure 1E**) (34). Meanwhile, the L452R mutation disrupts bamlanivimab binding since L452 is part of the epitope of bamlanivimab, forming a hydrophobic interaction with a leucine of bamlanivimab (**Figure 2D**). The positively charged arginine side chain would disrupt such interaction (**Figure 2E**), and as a result, L452R has 20-fold decreased affinity for bamlanivimab (**Table 1**) (20). L452R mutation has also been reported to escape the HLA-restricted T-cell immunity (34).

The spike threonine 478 is located on the edge of the receptor binding motif but does not directly contact ACE2 (17). The T478K mutation slightly increases ACE2 affinity due to a potential hydrogen bond formation between K478 and glutamine 24 of ACE2 (20). Although T478K does not affect bamlanivimab binding, this positively charged lysine mutation from threonine would change the binding of antibodies targeting this area.

#### Omicron Variant

**Figure 3:**
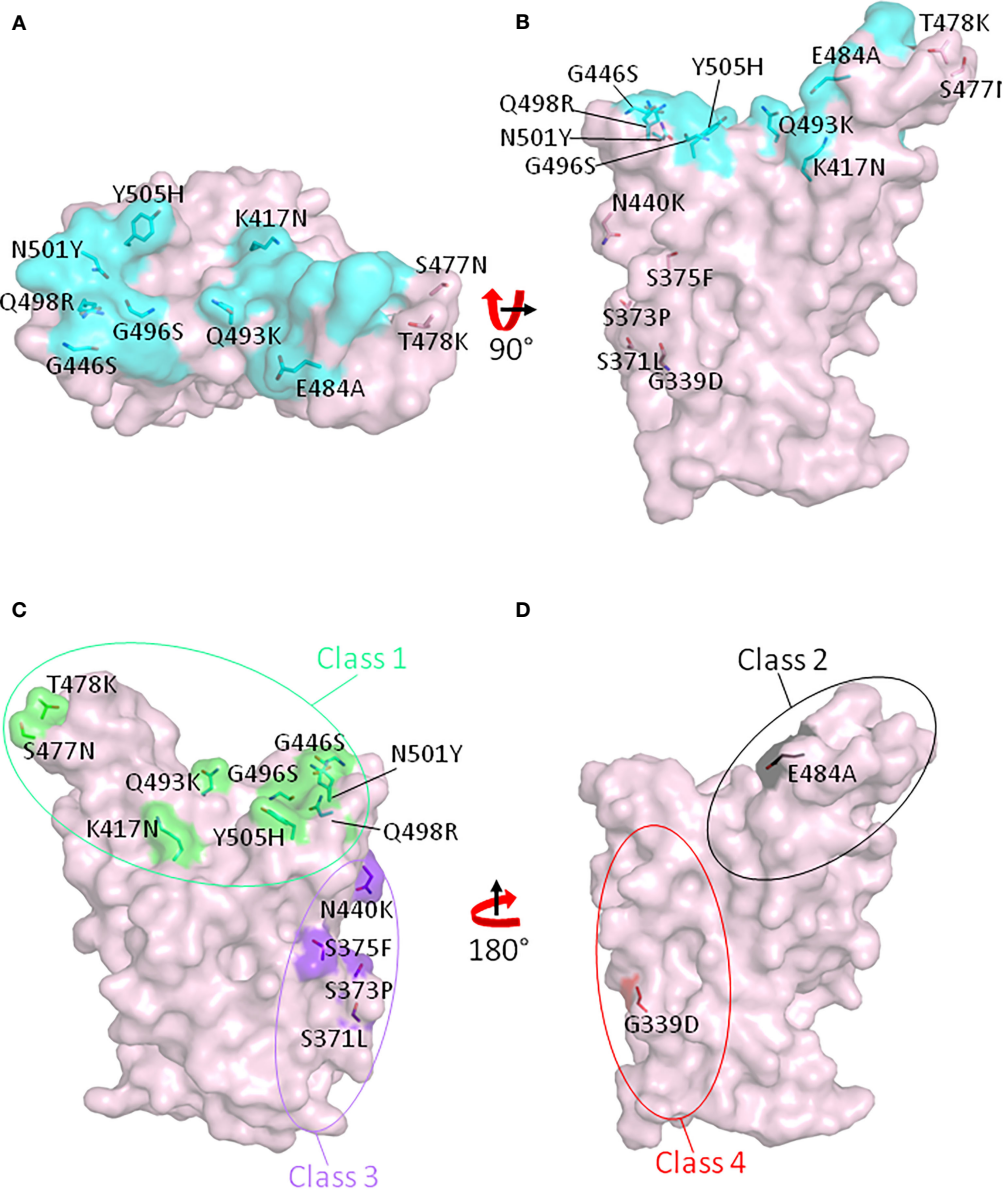
Mutated sites in the RBD region of the SARS-CoV-2 Omicron variant. Fifteen amino acids which have been mutated in the Omicron variant RBD are shown on the RBD with top view **(A)** and side view **(B)**. Cyan indicates the ACE2 footprint on the ancestral RBD. **(C)** Class 1 and class 3 neutralizing antibody binding spots on RBD are shown in green and purple, respectively. **(D)** Class 2 and class 4 neutralizing antibody binding spots on RBD are shown in black and red, respectively.

The Omicron variant is the most recent variant named as a VOC by the WHO. It was first identified in South Africa on November 11, 2021 and subsequently declared as a VOC on November 26, 2021. The major reason for declaring it as VOC at such short notice is the fact that the Omicron variant spike protein has 32 mutations, 15 of which are in the RBD region (**Table 1**). Importantly, 11 of 15 RBD mutations are located in the RBM, and 7 of these 11 mutations are contact residues with ACE2 (**Figures 3A, B****)**. Because the Omicron variation includes the RBD N501Y mutation, the Omicron variant RBD should have increased affinity for ACE2 as the other N501Y variants, Alpha, Beta, Gamma, and Mu, do. The Omicron E484A mutation should greatly decrease bamlanivimab binding due to the loss of salt bridge interactions between glutamic acid and arginines from the mAb. As expected, a recent research showed that bamlanivimab lost neutralization against the Omicron variant pseudovirus (35). Moreover, mutations in the Omicron RBD region span all four classes of neutralization hot spots (**Figure 3C, D****)**. These mutations also abolish the binding of casirivimab and imdevimab and escape more than 85% mAbs from a cohort of 247 tested mAbs targeting the RBD region (35). Compared with the Delta variant, authentic Omicron SARS-CoV-2 virus has been reported to reduce the mRNA-vaccinated serum antibody neutralization by 11- to 37-fold, depending on the status of vaccination (36).

### Variants of Interest

The SARS-CoV-2 variant of interest (VOI) is a variant with genetic changes that would increase its transmission or immune escape and causes community transmission (WHO SARS-CoV-2 Variants). As of November 2021, there are two variants of interest, Lambda and Mu.

#### Lambda Variant

The Lambda variant was first identified in December 2020 and is the dominant strain in Peru, South America (WHO SARS-CoV-2 Variants). This strain has two mutations in the RBD region, L452Q and F490S. The Lambda variant RBD has similar binding affinity to ACE2 as the ancestral RBD, but totally abolishes bamlanivimab binding (**Table 1**) (20). Pseudovirus with the Lambda spike decreased mRNA vaccinated serum neutralization by 2-fold (26).

As mentioned in the L452R mutation in the Delta variant, leucine 452 does not directly contact ACE2 (17). Unlike L452R, glutamine cannot form tighter electrostatic interactions with the negatively charged patch on ACE2, and therefore the L452Q mutation does not increase ACE2 affinity (20). L452Q mutation can also disrupt the hydrophobic interaction between the original RBD L452 and leucines from bamlanivimab (**Figure 2F**), although not as much as L452R. L452Q mutation decreased bamlanivimab affinity by 8-fold (20).

Phenylalanine 490 in the RBD region is also not a contact residue with ACE2 (17). The F490S mutation has no effect on ACE2 binding affinity at all. F490 is part of epitope for bamlanivimab, forming hydrophobic interactions with isoleucine from bamlanivimab (**Figure 2G**) (2). Mutation to polarized serine at this site would disrupt such interactions and therefore has been found to decrease the bamlanivimab affinity by 20-fold (**Figure 2H**). Together with L452Q mutation, F490S fully abolishes the bamlanivimab binding (20).

#### Mu Variant

Mu variant is the most recent variant of interest and the dominant strain in Colombia, South America (WHO SARS-CoV-2 Variants). Its RBD region has R346K, E484K, and N501Y mutations. The latter two mutations have also appeared in the Beta and Gamma variants. Since R346K is not in contact with ACE2 (17), this mutation should have no impact on ACE2 binding, while the change of the longer side chain and higher p*K*_a_ of arginine to the shorter and lower p*K*_a_ of lysine may affect immune escape. The Mu variant RBD increased ACE2 binding affinity by 9-fold, and E484K mutation can fully abolish bamlanivimab binding (**Table 1**). Pseudovirus harboring the Mu variant spike has been reported to decrease the antibody neutralization from mRNA-vaccinated serum by 10-fold, even higher than that of the Beta variant spike (26).

## Discussion

The SARS-CoV pandemic in 2003 was relatively easily confined because, although the virus itself was much more lethal than SARS-CoV-2, it was also less easily transmissible. The current SARS-CoV-2 pandemic has been much more serious because it induces less mortality than its predecessor; it is much more infectious and thereby greatly increases the numbers of hospitalized or dead. Although both viruses use the same human receptor, the 7-fold higher affinity of SARS-CoV-2 RBD than that of SARS-CoV for ACE2 makes SARS-CoV-2 much more contagious (17). With hundreds of millions of infections, SARS-CoV-2 virus had solid ground to evolve and mutate resulting in the appearance of many variants, including those now identified as VOI or VOC. In this review, we focus on the effects of SARS-CoV-2 RBD mutations from VOC and VOI on ACE2 binding and antibody escape, using bamlanivimab as a model. We combined experimental affinity measurement with structural modeling to elucidate the consequences of each mutation for ligand binding and antibody escape.

As tools in combating virus infections, host antibodies are made against any foreign antigen including viruses. During virus infection or vaccination, the antigen-specific B cells go through affinity maturation and class switching (37). The antibodies are secreted to clear the virus. It may be operated by promoting uptake and destruction of the virus by macrophages and other phagocytic cells mediated *via* Fc receptors and/or complement fixation (38). Antibodies can also neutralize the virus directly by inhibiting the ability of the virus to bind its ligand and infect cells, in the case of SARS-CoV-2 this process involves, among others, binding of the spike protein RBD to host ACE2. The assays usually used to assess the activity of anti-SARS-CoV-2 involve direct measurement of antibody levels *via* ELISAs or measurement of virus neutralization by prevention of infection of target cells *in vitro*. Neither of these methods measure *in toto*, all the ways in which antibody might affect the virus, although they are probably pretty good measures of the relative efficiency of the antisera. Also to be considered here are the relative efficiencies of CD4 and CD8 T cells specific for the virus.

With these points in mind, the host immune response is certainly a source of selection pressure for the virus to mutate, and this can be done quite easily by the virus. It only took, for example, one amino acid switch to abolish the elite therapeutic antibody bamlanivimab in the case of E484K mutation (19). Luckily, the mutation rate of SARS-CoV-2 is relatively low due to its RdRp-independent proof-reading activity (12). Nevertheless, the fact that there are at least 330,000 new infections by the virus each day, worldwide (worldometers.info/coronavirus) means that it is fertile ground for mutants to appear, even given the fidelity of its RdRp. Still, it is expected that a portion of the memory B cells against the ancestral RBD could also react with the RBD variants (27), at least, so far, although this remains to be seen with respect to the new Omicron variant.

## Author Contributions

HL wrote a draft of the manuscript. PW and HL prepared the figures. JK, PM, and GZ gave suggestions and edited the manuscript.

## Conflict of Interest

HL was partially supported by NB Life Laboratory LLC at the beginning of the research.

The remaining authors declare that the research was conducted in the absence of any commercial or financial relationships that could be construed as a potential conflict of interest.

## Publisher’s Note

All claims expressed in this article are solely those of the authors and do not necessarily represent those of their affiliated organizations, or those of the publisher, the editors and the reviewers. Any product that may be evaluated in this article, or claim that may be made by its manufacturer, is not guaranteed or endorsed by the publisher.
